# Identification of a p53-repressed gene module in breast cancer cells

**DOI:** 10.18632/oncotarget.19608

**Published:** 2017-07-26

**Authors:** Takafumi Miyamoto, Chizu Tanikawa, Varalee Yodsurang, Yao-Zhong Zhang, Seiya Imoto, Rui Yamaguchi, Satoru Miyano, Hidewaki Nakagawa, Koichi Matsuda

**Affiliations:** ^1^ Laboratory of Genome Technology, Human Genome Center, Institute of Medical Science, The University of Tokyo, Tokyo, Japan; ^2^ Laboratory of DNA Information Analysis, Human Genome Center, Institute of Medical Science, The University of Tokyo, Tokyo, Japan; ^3^ Division of Health Medical Data Science, Health Intelligence Center, Institute of Medical Science, The University of Tokyo, Tokyo, Japan; ^4^ Laboratory for Genome Sequencing Analysis, RIKEN Center for Integrative Medical Sciences, Tokyo, Japan; ^5^ Laboratory of Clinical Genome Sequencing, Department of Computational Biology and Medical Sciences, Graduate School of Frontier Sciences, The University of Tokyo, Tokyo, Japan

**Keywords:** p53, breast cancer, transcriptome analysis, adriamycin, gene module

## Abstract

The p53 protein is a sophisticated transcription factor that regulates dozens of target genes simultaneously in accordance with the cellular circumstances. Although considerable efforts have been made to elucidate the functions of p53-induced genes, a holistic understanding of the orchestrated signaling network repressed by p53 remains elusive. Here, we performed a systematic analysis to identify simultaneously regulated p53-repressed genes in breast cancer cells. Consequently, 28 genes were designated as the p53-repressed gene module, whose gene components were simultaneously suppressed in breast cancer cells treated with Adriamycin. A ChIP-seq database showed that p53 does not preferably bind to the region around the transcription start site of the p53-repressed gene module elements compared with that of p53-induced genes. Furthermore, we demonstrated that p21/CDKN1A plays a pivotal role in the suppression of the p53-repressed gene module in breast cancer cells. Finally, we showed that appropriate suppression of some genes belonging to the p53-repressed gene module contributed to a better prognosis of breast cancer patients. Taken together, these findings disentangle the gene regulatory network underlying the built-in p53-mediated tumor suppression system.

## INTRODUCTION

Breast cancer is the most common cancer in females worldwide. Accumulating evidence has revealed that many genes are involved in the carcinogenesis of breast cancer. A loss of heterozygosity in chromosomes 1, 3p, 6q, 7q, 8, 9p, 10q, 11q, 13q, 16q, 17, 18q, 20q, 22q, or X has been detected as genetic abnormalities in breast cancers [1, 2]. In addition, several genetic syndromes, including hereditary breast and/or ovarian cancer syndrome (caused by *BRCA1* and *BRCA2* mutations) [3]], Cowden syndrome (caused by *PTEN* gene mutation) [4, 5], hereditary diffuse gastric cancer (caused by *CDH1* gene mutation) [6], Li-Fraumeni syndrome (caused by *p53* gene mutation) [7], and Peutz-Jeghers syndrome (caused by *STK11* gene mutation) [8], have been reported to increase the risk of breast cancer. Among those genes whose germline mutation predisposes a person to breast cancer, the transcription factor gene *p53* is the second most frequently mutated gene (24% in the Catalog of Somatic Mutations in Cancer (COSMIC) database) after *PIK3CA* (27% in COSMIC) in breast cancer [9]. Thus, p53 is one of the key molecules that prevent the development of breast cancer as well as other types of cancer.

The p53 protein is at the core of the signaling network that governs the cell-intrinsic tumor suppression system. Enormous research efforts have revealed the diversity of p53-regulated cellular functions (e.g., cell cycle arrest, apoptosis, senescence, and metabolic reprogramming) [10]. Befitting its role in maintaining cellular fitness by regulating these cellular functions, p53 induces an appropriate set of target genes in response to cellular stressors. To date, over one hundred genes have been reported to be transactivated by p53 (p53-induced genes) [11]. In contrast, genes whose expression is suppressed by p53 (p53-repressed genes) are poorly known. Indeed, p53-repressed genes account for less than 20% of the p53 targetome that has been reported [11]. Because p53 mediates the transactivation of many target genes in accordance with their intrinsic function, it is a fascinating avenue of p53 research to accumulate knowledge on p53-repressed genes and their cellular functions. In addition, it is important to understand the intrinsic gene regulatory mechanism, particularly the set of genes that is coexpressed as a gene module to facilitate p53-regulated cellular processes appropriately under specific conditions.

In this study, we identified 44 genes as p53-repressed genes in breast cancer using a combination of three different transcriptome analyses. Among them, 28 genes were classified into the p53-repressed gene module, whose gene elements were suppressed simultaneously in response to genotoxic stress in breast cancer cells. Many of p53-repressed genes are involved in cell cycle regulation. In addition, we found that p53 suppressed the expression of these genes, at least in part, *via* the p21/CDKN1A-mediated system. Finally, we showed that downregulation of p53-repressed genes is associated with the favorable prognosis of breast cancer.

## RESULTS

### Identification of p53-repressed gene candidates

To comprehend the gene network repressed by p53, we utilized three independent sets of transcriptome analyses: (i) microarray of Adriamycin (ADR)-treated *p53* knockout MCF10A breast epithelial cells (MCF10A *p53*−/−) and their wild-type counterparts (MCF10A *p53*+/+) (MCF10A cells dataset, Supplementary Table S1); (ii) high-throughput RNA sequencing (RNA-seq) data from the mammary gland of X-ray-irradiated *p53*−/− mice and genetically matched *p53*+/+ mice (*p53* mice dataset, Supplementary Table S2) [50]; and (iii) RNA-seq of breast invasive carcinoma harboring wild-type *p53* (*p53*WT) and *p53* mutations (*p53*Mt) obtained from the Cancer Genome Atlas (TCGA) database (TCGA-BRCA) [12] (TCGA dataset, Supplementary Table S3).

**Figure 1:**
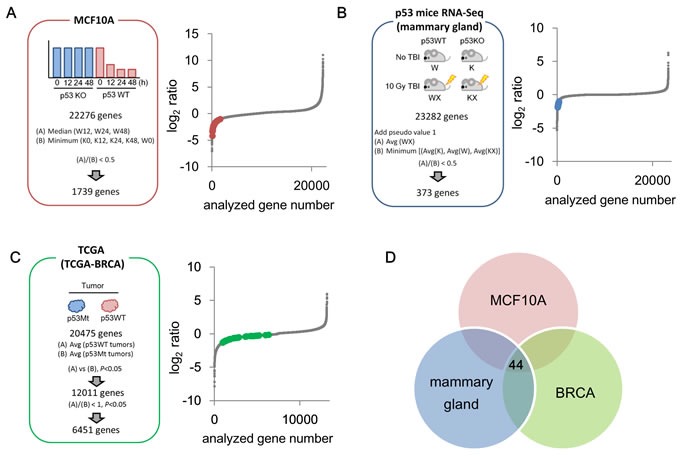
Identification of p53-repressed gene candidates **A.** Schematic of the MCF10A cells dataset screen is shown. MCF10A *p53^+/+^* cells and MCF10A *p53^−/−^* cells were treated with 0.5 μg/ml ADR for 2 hours, then cultured with fresh medium by indicated time. Subsequently, the cells were subjected to transcriptome analysis. Red dots indicate 44 common p53-repressed gene candidates identified in (D). W: MCF10A *p53^+/+^* cells, K: MCF10A *p53^−/−^* cells. **B.** Schematic of the *p53* mice dataset screen is shown. *p53^+/+^* and *p53^−/−^* female mice (10 weeks old) were irradiated at 10 Gy (total body irradiation [TBI]). Twenty-four hours after irradiation, the gene expression level in the mammary gland was analyzed using RNA-seq. Blue dots indicate 44 common p53-repressed gene candidates identified in (D). **C.** Schematic of the TCGA dataset (BRCA: Breast Invasive Carcinoma) screen is shown. Green dots indicate 44 common p53-repressed gene candidates identified in (D). **D.** Venn diagram displaying the overlap between the three datasets obtained from **A.**, **B.**, and **C.**

To identify p53-repressed genes, we set a discrimination criterion for each dataset (Figure 1A-1C). As a result, 1,739 genes, 373 genes, and 6,451 genes were identified as candidates for p53-repressed genes in the MCF10A cells dataset, *p53* mice dataset, and TCGA dataset, respectively (Figure 1A-1C). We subsequently combined these datasets to detect p53-repressed genes with higher fidelity. Consequently, 44 genes were selected as p53-repressed gene candidates (Figure 1D, Supplementary Figure S1). Among them, 17 genes (*AURKB* [13], *BIRC5* [14], *CCNA2* [15], *CCNB1* [15], *CCNB2* [16], *CDC20* [17]*, CDCA8* [18]*, CENPA* [19]*, CEP55* [20]*, KIF23* [21]*, LMNB1* [22]*, MCM5* [23]*, PLK1* [24]*, RACGAP1* [25]*, RRM2* [26]*, TOP2A* [27], and *UBE2C* [28]) have been reported as being p53-repressed genes with experimental verification.

### Identification of a p53-repressed gene module in breast cancer cells under genotoxic conditions

Because p53 can regulate the expression of multiple genes concurrently, it is important to understand a set of genes that are co-regulated under specific conditions [29]. Thus, we tried to detect a p53-repressed gene module that responded to genotoxic stress in breast cancer cells. For this purpose, we performed quantitative PCR (qPCR) using seven breast cancer cell lines. Four cell lines (HBL-100, HBC4, MCF-7, and ZR-75-1) had wild-type p53, whereas the others (T-47D, SK-BR-3, and BT-549) harbored a p53 mutation. ADR treatment induced major p53 targets, *p21/CDKN1A* and *MDM2* mRNA, in wild-type p53 cells, whereas the mRNA expression levels were maintained at low levels in cancer cells harboring a p53 mutation, regardless of the ADR treatment (Supplementary Figure S2A). Taken together, these results indicated that the transactivation activity of p53 was inactivated in cancer cells that have mutant p53.

**Figure 2:**
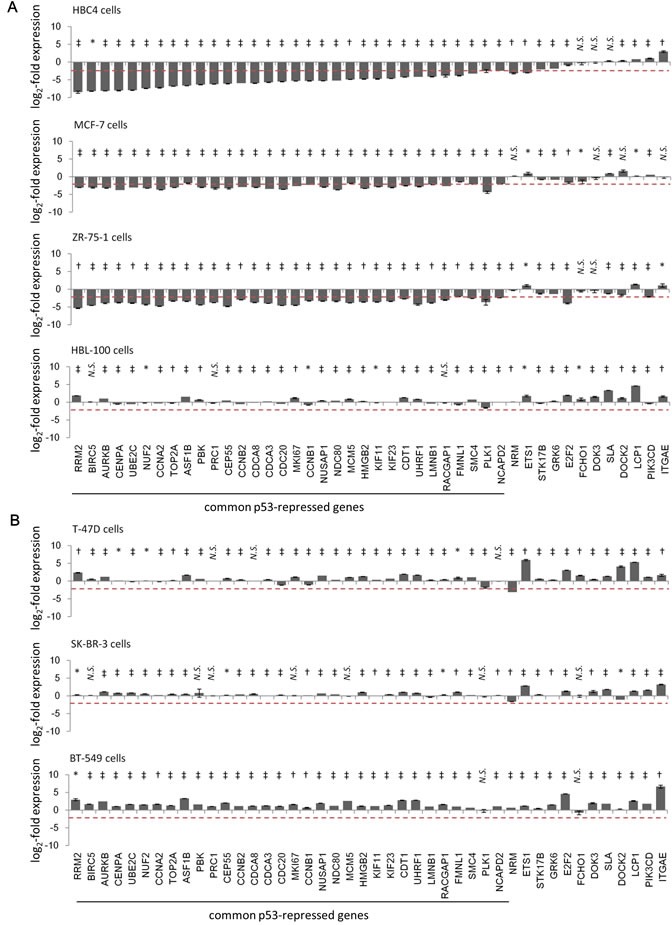
Identification of common p53-repressed genes in ADR-treated breast cancer cells by ADR treatment Indicated breast cancer cells [(**A**) cells harboring wild-type p53, (**B**) cells harboring p53 mutations] were treated with 2 μg/ml ADR for 2 hours. Forty-eight hours after treatment, the mRNA expression level of the indicated genes was determined using qPCR and the log_2_-fold change of mRNA expression from ADR-treated compared to untreated cells were calculated using the ΔΔCt method. The graph shows the log_2_-fold change of mRNA expression in ADR-treated cells. The data are presented as the mean±SD from three independent experiments. The red dotted line shows the cutoff value. *P < 0.05, †*P* < 0.01, ‡*P* < 0.001, N.S., not statistically significant.

Next, from the 44 p53-repressed gene candidates (Figure 1D), we selected the genes whose expression level was diminished after ADR treatment in at least two of the four cell lines with wild-type p53, but not in the three cell lines that harbored the p53 mutation. A log2-fold change value ≤ -2 was a criterion for a gene to be considered as repressed by ADR treatment. Consistent with the qPCR results, we selected 32 genes as candidates for p53-repressed genes that displayed a log2-fold change value of ≤ -2 in ADR-treated breast cancer cell lines (Figure 2). PLK1, one of the p53-repressed gene products, exhibited similar expression dynamics in terms of mRNA and protein levels, indicating the validity of our screening methods (Supplementary Figure S3). In the case of HBL-100 cells, ADR treatment did not repress any of the 44 genes, although *p21/CDKN1A* and *MDM2* mRNA was strongly upregulated after ADR treatment (Supplementary Figure S2A). One potential reason is that HBL-100 cells expressed the SV40 viral antigen [30], which might have affected p53-mediated gene repression [31].

**Figure 3:**
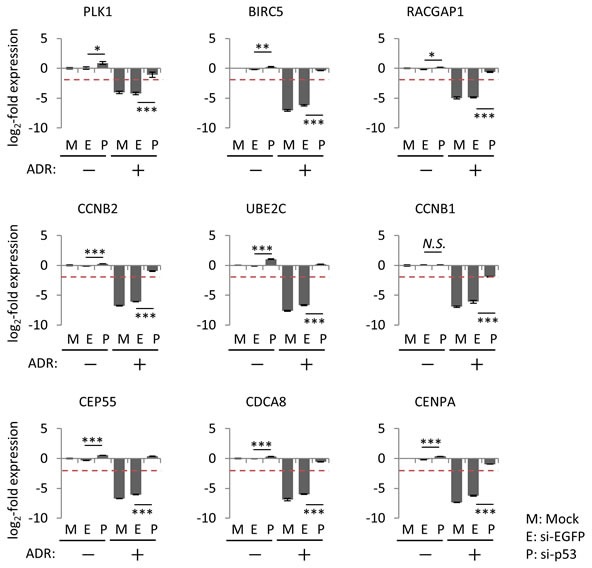
Effect of p53 knockdown on the regulation of common p53-repressed genes At 24 hours after transfection of the indicated siRNA, HBC4 cells were treated with 2 μg/ml ADR for 2 hours. Forty-eight hours after treatment, the mRNA expression level of the indicated genes was determined using qPCR and the log2-fold change of mRNA expression against the control condition (Mock, no ADR treatment) was calculated using the ΔΔCt method. The data are presented as the mean±SD from three independent experiments. The red dotted line shows cutoff value. *P < 0.05, **P < 0.01, ***P < 0.001, *N.S.*, not statistically significant. Representative data (9 genes) are presented. Additional data are shown in Supplementary Figure S4.

**Figure 4:**
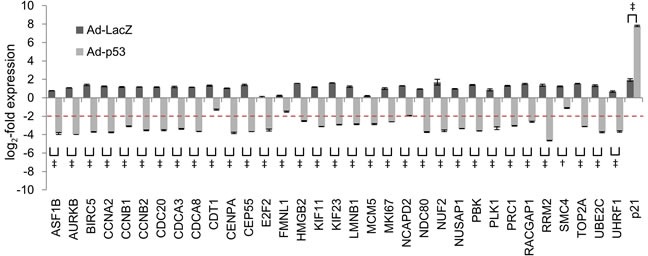
Effect of Ad-p53 on the regulation of common p53-repressed genes T-47D cells were infected with adenoviruses expressing either LacZ (Ad-LacZ) or wild-type p53 (Ad-p53) at multiplicities of infection (MOI) of 40. At 48 hours after transduction, the mRNA expression level of indicated genes was determined using qPCR and the log_2_-fold change of mRNA expression against the control condition (no adenovirus transduced cells) were calculated using the ΔΔCt method. The graph shows the log_2_-fold change of mRNA expression in Ad-LacZ-transduced cells and Ad-p53-transduced cells. The data are presented as the mean±SD from three independent experiments. The red dotted line shows the cutoff value. †*P* < 0.01, ‡*P* < 0.001.

To determine whether these 32 genes were repressed by ADR treatment in a p53-dependent manner, we performed siRNA-mediated *p53* knockdown in HBC4 cells harboring wild-type *p53*. Knockdown of *p53* resulted in the suppression of *p21/CDKN1A* mRNA expression, which indicated that p53 function was perturbed in *p53*-knockdown cells (Supplementary Figure S2B). Consistent with this finding, p53 knockdown completely abrogated ADR-induced suppression of all 32 genes (Figure 3 and Supplementary Figure S4). Next, we examined the effect of the transduction of adenovirus expressing wild-type p53 (Ad-p53) into T-47D cells harboring a p53 mutation. The log2-fold change value ≤ -2 was a criterion for a gene to be considered as repressed by exogenous p53. Ad-p53 transduction revealed that the expression of 28 of the 32 genes was repressed by the ectopic expression of p53 (Figure 4). Overall, we identified 28 genes that were simultaneously suppressed in breast cancer cells exposed to genotoxic stress (hereafter referred to as the p53-repressed gene module). Gene ontology analysis by DAVID Functional Annotation tool [32] revealed that the p53-repressed gene module was mainly assigned to regulation of the cell cycle (Supplementary Figure S5).

### Examination of gene expression dynamics of the p53-repressed gene module across tissues

**Figure 5:**
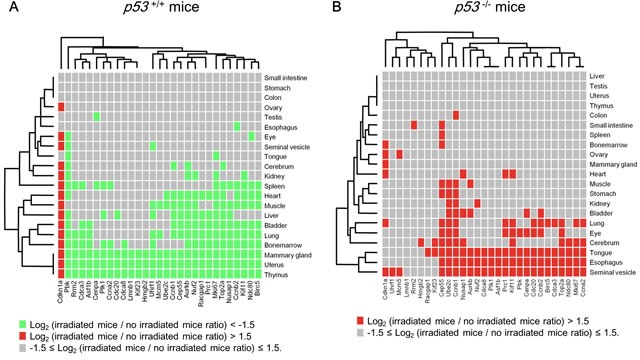
Systemic regulation of the p53-repressed gene module **A.** *p53^+/+^* mice were irradiated at 10 Gy (total body irradiation). Twenty-four hours after irradiation, the change in the indicated gene expression level was calculated as the ratio of irradiated *p53^+/+^* mice to non-irradiated *p53^+/+^* mice. Each tissue, *n* = 3 (mammary gland and ovary, *n* = 2). **B.** *p53^−/−^* mice were irradiated at 10 Gy (total body irradiation). Twenty-four hours after irradiation, the change in the indicated gene expression level was calculated as the ratio of irradiated *p53^−/−^* mice to non-irradiated *p53^−/−^* mice. Each tissue, *n* = 3 (mammary gland and ovary, *n* = 2).

Although p53 is ubiquitously expressed, the set of genes transactivated by p53 differed across tissues [33]. To determine whether the p53-repressed gene module identified in breast cancer cells is a finite module or a tissue-specific one under genotoxic conditions, we analyzed the RNA-seq dataset of various tissues of X-ray-irradiated *p53*−/− mice and genetically matched *p53*+/+ mice [50]. Multi-tissue analysis revealed that the p53-repressed gene module elements were suppressed in many tissues of irradiated *p53*+/+ mice compared with non-irradiated *p53*+/+ mice (Figure 5A). Importantly, simultaneous suppression of the p53-repressed gene module was observed in the mammary gland, uterus, and thymus but not in other tissues in irradiated-*p53*+/+ mice (Figure 5A), indicating that the regulation of the p53-repressed gene module was highly tissue-specific. In contrast, simultaneous suppression of the p53-repressed gene module was not observed in *p53*−/− mice (Figure 5B).

### Analysis of the occupancy of p53 around the TSS of p53-repressed gene module elements

**Figure 6:**
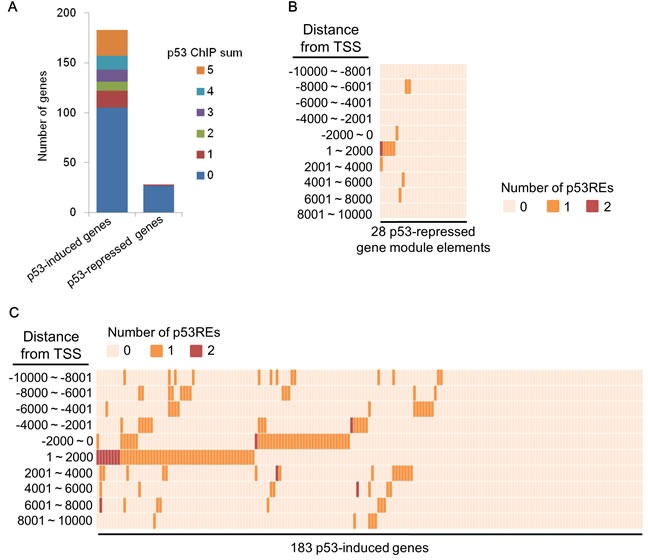
p53REs around TSS of p53-mediated genes **A.** The number of p53 response elements (p53REs) in the promoter region of p53-mediated genes obtained from five different ADR-treated cells datasets in the database on human target gene regulation in the p53 and cell cycle networks is shown. **B.** The number of p53REs around TSS of p53-repressed gene module obtained from ReMap database is shown. **C.** The number of p53REs around TSS of p53-induced genes obtained from the ReMap database is shown.

In response to various cellular stressors, p53 binds to the consensus motif (p53 response element, p53RE) around target genes, resulting in the induction of p53-induced genes [11]. However, the requirement of p53 binding to p53REs or a region around the transcription start site (TSS) for p53-mediated suppression remains a controversial topic [34, 35]. To test whether the p53-repressed gene module contained p53-binding regions around the TSS, we assessed p53 ChIP-seq datasets obtained using cells that had been treated with ADR (http://www.targetgenereg.org/) [36]. For this analysis, 183 previously reported genes were classified into p53-induced genes (Supplementary Table S4). Five different p53 ChIP-seq datasets in the database showed that p53 preferably bound to a promoter region (±2 kb from a TSS) of p53-induced genes (78 genes, 42.6%) rather than the p53-repressed gene module (1 gene, 3.7%) (Figure 6A), indicating that p53-induced genes were significantly enriched for p53REs (*p* = 0.0000154, Fisher’s exact test). To further examine the prevalence of p53REs around the TSS of p53-induced genes, we examined p53 ChIP-seq datasets in the public database ReMap [37]. Analysis of the promoter region of p53REs showed that 46.4% (85 genes) of the p53-induced genes and 21.4% (6 genes) of the p53-repressed gene module contained p53REs (*p* = 0.0139, Fisher’s exact test) (Figure 6B and 6C). Similar results were also observed in a region within ±10 kb from the TSS of p53-induced genes (63.4%, 116 genes) and p53-repressed gene module (35.7%, 10 genes) (*p* = 0.007, Fisher’s exact test) (Figure 6B and 6C). Taken together, these results suggested that p53 could regulate the p53-repressed gene module *via* an indirect mechanism.

### p21/CDKN1A is a key molecule for regulating the p53-repressed gene module

**Figure 7:**
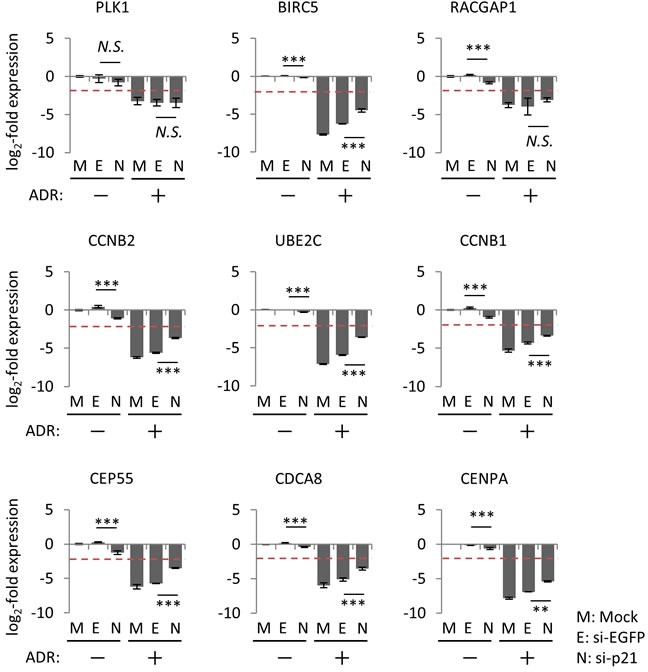
Effect of p21/CDKN1A knockdown on the regulation of p53-repressed gene module At 24 hours after transfection of indicated siRNA, HBC4 cells were treated with 2 μg/ml ADR for 2 hours. Forty-eight hours after treatment, the mRNA expression level of the indicated genes was determined using qPCR, and the log2-fold change of mRNA expression against the control condition (Mock, no ADR treatment) was calculated using the ΔΔCt method. The data are presented as the mean±SD from three independent experiments. The red dotted line shows the cutoff value. **P < 0.01, ***P < 0.001, *N.S.*, not statistically significant. Representative data (9 genes) are presented. Additional data are shown in Supplementary Figure S6.

Although *p21/CDKN1A* is the preferred target gene of p53, accumulating evidence indicated that p21/CDKN1A plays a pivotal role in p53-mediated repression of various downstream target genes [15, 38–40]. To determine whether p21/CDKN1A is required for the suppression of the p53-repressed gene module, *p21/CDKN1A* in HBC4 cells was silenced using small interfering RNA (siRNA). We found that knockdown of *p21/CDKN1A* attenuated the ADR-induced suppression of the p53-repressed gene module compared to control cells treated with siEGFP (Figure 7 and Supplementary Figure S6), which suggested that p21/CDKN1A was, at least partially, involved in the regulation of the p53-repressed gene module. Of note, knockdown of *p21/CDKN1A* had no effect on the ADR-induced suppression of *PLK1* and *RACGAP1* in HBC4 cells (Figure 7).

**Figure 8:**
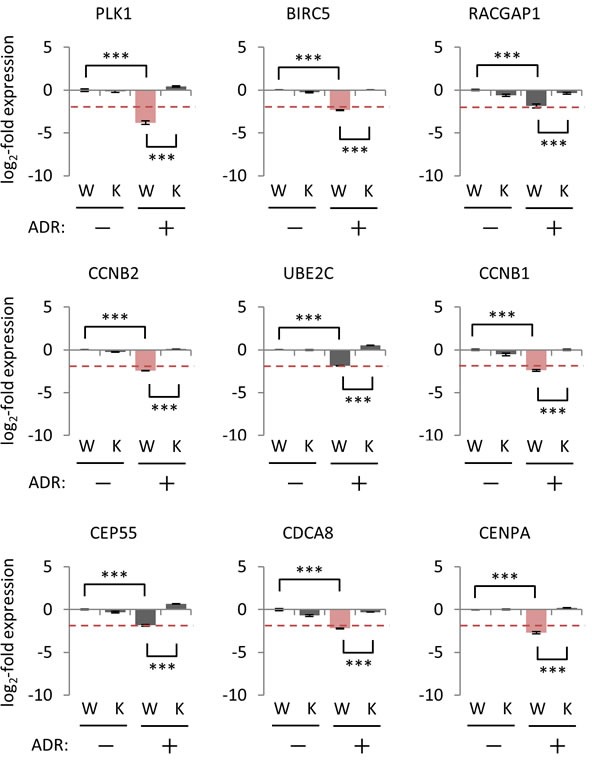
Effect of p21/CDKN1A knockout on the regulation of p53-repressed gene module HCT116 *p21^+/+^* cells (W) and *p21^−/−^* cells (K) were treated with 2 μg/ml ADR for 2 hours. Forty-eight hours after treatment, the mRNA expression level of the indicated genes was determined using qPCR, and the log2-fold change of mRNA expression against the control condition (HCT116 *p21^+/+^* cells, no ADR treatment) was calculated using the ΔΔCt method. The data are presented as the mean±SD from three independent experiments. The red dotted line shows the cutoff value. Red graph: lower than cutoff value. ***P < 0.001, *N.S.*, not statistically significant. Representative data (9 genes) are presented. Additional data are shown in Supplementary Figure S7.

To further examine the effect of p21/CDKN1A on the regulation of p53-repressed gene module, colorectal cancer HCT116 *p21*−/− cells and their wild-type counterparts were subjected to qPCR analysis. Although HCT116 *p21*−/− cells retained the genotoxic stress-induced transactivation activity of p53 as measured by the *FAS* levels, which is a major p53 target gene [41], the expression level of *p21/CDKN1A* mRNA was abrogated (Supplementary Figure S7). Under genotoxic stress, we found that 17 of the 28 p53-repressed gene module (*AURKB, BIRC5, CCNA2, CCNB1, CCNB2, CDC20, CDCA3, CDCA8, CENPA, HMGB2, KIF11, LMNB1, MKI67, NDC80, NUF2, PLK1*, and *PRC1*) were suppressed in HCT116 *p21*+/+ cells (Figure 8 and Supplementary Figure S7). Suppression of all 17 genes was disinhibited in ADR-treated HCT116 *p21*−/− cells compared to ADR-treated HCT116 *p21*+/+ cells (Figure 8 and Supplementary Figure S7). Importantly, the effect of p21 perturbation on the ADR-induced suppression of p53-repressed gene module differed between HBC4 cells and HCT116 *p21*−/− cells. Specifically, genotoxic stress-induced *PLK1* suppression was significantly disinhibited in HCT116 *p21*−/− cells, but not in *p21/CDKN1A*-knockdown HBC4 cells (Figures 7 and 8). Taken together, these results suggested that the mechanism of p53-mediated suppression for a specific gene (e.g. *PLK1*) is different among cell types and/or tissues.

### The importance of the p53-repressed gene module for the prognosis of breast cancer

**Figure 9:**
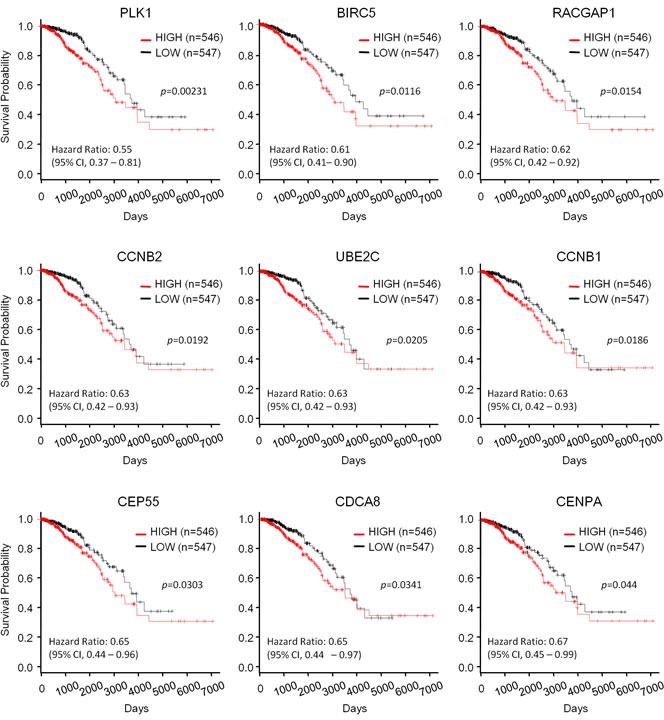
The relationship between the expression level of p53-mediated genes and prognosis of breast cancer patients Kaplan-Meier survival curves of the higher expression group and lower expression group for the indicated genes. *P*-values were calculated using the log-rank test.

In breast cancer, p53 mutations are associated with worse overall survival [42]. We assessed whether p53-repressed gene module affects the prognosis of breast cancer. Breast cancer patients in the TCGA dataset were subdivided into two groups: a higher expression group (High) and a lower expression group (Low) of the corresponding p53-repressed gene module. The median mRNA expression level was used as the cutoff value. As a result, we found that 9 of the 28 p53-repressed gene module correlated with survival outcome among breast cancer patients in a statistically significant manner (Figure 9). Fisher’s exact test of independence confirmed that mutant p53 was significantly associated with the elevated expression of the 9 p53-repressed genes (Supplementary Figure S8). These results suggested that p53 mutations affected the patients’ prognosis *via* perturbation of the p53-repressed gene module.

## DISCUSSION

To date, some breast cancer predisposition genes have been identified [43]; among them, the transcription factor *p53* is commonly mutated in both breast cancers and other cancers [1, 44]. Thus, the rationale for developing attractive breast cancer therapies that target p53 is to reconstitute the most crucial p53 signaling network, which is indispensable for the operation of the p53-regulated tumor suppression system. To achieve this goal, we focused on the identification of p53-repressed genes because potential p53-repressed genes remain poorly known compared with p53-induced genes [11]. Consequently, we identified 44 genes as common p53-repressed genes. Among these, 28 genes (including 17 previously reported p53-repressed genes) were identified as the p53-repressed gene module that respond to genotoxic stress simultaneously in breast cancer cells, as verified by qPCR. GO analysis indicated that these genes are involved in the regulation of various cellular functions in a coordinated manner, suggesting that simultaneity is a key concept in the orchestration of p53 functions under particular conditions.

Notably, 4 genes (CDT1, FMNL1, NCAPD2, and SMC4) were not suppressed by overexpression of wild-type p53 (Figure 4), despite having exhibited p53-dependent repression in response to genotoxic stress (Supplementary Figure S4). One potential reason is that the p53 mutant inhibited wild-type p53-mediated repression of these genes. Future studies may reveal the negating effects of the p53 mutants [45] on wild-type p53-mediated gene repression.

Simultaneous suppression of the 28 gene elements was also observed in previously reported database [36], including Nutlin-3a-treated MCF-7 breast cancer cells, HepG2 liver cancer cells, IMR90 fetal lung fibroblasts, and HCT116 colorectal cancer cells as well as ADR-treated GM00011 fetal skin fibroblasts. These results suggested that the p53-repressed gene module identified in this study might function as a core module of p53-repressed genes under various conditions and/or cell lines. Of note, 18 of the 28 p53-repressed genes were assigned as essential genes for optimal cell proliferation in chronic myelogenous leukemia cell lines [46]. Thus, fine-tuning and coordination of expression of each gene may be required to appropriately execute p53-mediated cellular functions. Future studies will reveal which p53 target genes are transactivated under a specific condition as the p53-mediated gene module and which p53-mediated gene module is a prerequisite to execute p53-regulated cellular functions.

It is well established that p53 transactivates the expression of p53-induced genes by binding to p53REs near the TSS of target genes [36]. However, the regulatory mechanisms of p53-mediated repression have little in common with the mechanisms of p53-mediated transactivation. Indeed, there are various mechanisms by which p53 represses the expression of its downstream targets; these mechanisms can be divided into two categories: p53-dependent or p21/CDKN1A-dependent repression systems [34, 47]. In the p53-dependent repression system, p53 competes with other transcription factors or functions as a scaffold protein to recruit chromatin-remodeling factors to modify histone methylation status [47]. In addition, p53 promotes DNA methylation and inhibits the expression of large families of interspersed and tandem repeats [48]. However, the regulatory mechanisms of p53-dependent repression systems remain to be determined. As an alternative to the p53-dependent repression systems, computational meta-analysis of genome-wide data revealed that p21/CDKN1A-dependent repression system (e.g. the p53-p21-DREAM/RB pathway) is a core system or is potentially the only system to suppress the expression of downstream p53-repressed genes [35]. Indeed, we also demonstrated that p21/CDKN1A plays a pivotal role in suppressing the expression of the p53-repressed gene module in breast cancer cells. However, we found that p21/CDKN1A may not be the only node connecting p53 and p53-repressed genes. For example, suppression of *PLK1* did not disinhibit in *p21/CKDN1A*-silenced HBC4 cells under genotoxic stress (Figure 7), whereas the *PLK1* suppression was obviously disinhibited in HCT116 *p21*−/− cells (Figure 8). Thus, we hypothesized that the regulatory mechanism of p53-repressed genes is different among cell types even for the same gene. Consistent with these findings, our p53 mouse dataset indicated that the regulation of the p53-repressed gene module showed a tissue-specific pattern. Further experiments are necessary to uncover the molecular mechanism underlying the repression of p53 downstream genes.

In conclusion, we identified 28 p53-repressed genes that were simultaneously suppressed in breast cancer cells under genotoxic stress (Supplementary Figure S9). The p53-repressed gene module in breast cancer cells is, at least partially, in the p21/CDKN1A-dependent suppression system. Furthermore, multi-tissue analysis of the p53-repressed gene module showed that the extent of suppression varied among tissues as well as genes. Our findings provide insight into a novel gene regulatory network to disentangle a complex p53 signaling network under both physiological and pathological conditions. In addition, a precise understanding of a p53-regulated gene module may eventually lead to profound insights into the treatment and/or cure of cancers where a p53-regulated gene module has gone awry.

## MATERIALS AND METHODS

### Antibodies

Anti-PLK1 antibody (sc-53751) was purchased from Santa Cruz Biotechnology. Anti-p53 (OP43) and anti-p21 (OP64) antibodies were obtained from MERCK MILLIPORE.

### Cell culture and treatment

Human breast cancer cell line HBC4 cells were a kind gift from Dr. T. Yamori (The Cancer Institute of JFCR). HBL-100, MCF-7, ZR-75-1, T-47D, SK-BR-3, and BT-549 human breast cancer cell lines were purchased from the American Type Culture Collection. HCT116 *p21*+/+ and HCT116 *p21*−/− cells lines were gifts from Prof. B. Vogelstein (Johns Hopkins University, USA). MCF10A *p53*+/+ cells and MCF10A *p53*−/− cells were purchased from Sigma-Aldrich. HBL-100 cells were cultured in Dulbecco’s modified Eagle’s medium (DMEM; Gibco) supplemented with 10% fetal bovine serum (FBS) and 1% penicillin/streptomycin at 37°C in 5% CO2. HBC4, SK-BR-3, T-47D, BT-549, and ZR-75-1 cells were cultured in RPMI 1640 (Gibco) supplemented with 10% FBS and 1% penicillin/streptomycin at 37°C in 5% CO2. MCF-7 cells were cultured in minimum essential medium (MEM; Gibco) supplemented with 10% FBS, 0.01 mg/ml bovine insulin, Non-Essential Amino Acids Solution (Gibco, cat# 11140-050), sodium pyruvate (Gibco, cat#11360-070), and 1% penicillin/streptomycin at 37°C in 5% CO2. MCF10A *p53*+/+ cells and MCF10A *p53*−/− cells were cultured in MEGM mammary epithelial cell growth medium (LONZA) supplemented with 100 ng/ml cholera toxin at 37°C in 5% CO2. HCT116 *p21*+/+ and HCT116 *p21*−/− cells were cultured in McCoy’s 5a medium (Gibco) supplemented with 10% FBS and 1% penicillin/streptomycin at 37°C in 5% CO2. Small interfering RNA (siRNA) oligonucleotides, commercially synthesized by Sigma Genosys, were transfected with Lipofectamine RNAiMAX reagent (Invitrogen). Sequences of siRNA oligonucleotides are as follows. Si-EGFP: forward, 5′-GCAGCACGACUUCUUCAAG-3′; reverse, 5′-CUUGAAGAAGUCGUGCUGC-3′. Si-p53: forward, 5′-GACUCCAGUGGUAAUCUAC-3′; reverse, 5′-GUAGAUUACCACUGGAGUC-3′. Si-p21/CDKN1A: forward, 5′- GAUGGAACUUCGACUUUGU-3′; reverse, 5′-ACAAAGUCGAAGUUCCAUC-3′. We generated and purified replication-deficient recombinant viruses expressing p53 (Ad-p53) or LacZ (Ad-LacZ) as previously described [49]. T-47D cells were infected with viral solutions at various multiplicities of infection (MOI) and incubated at 37°C until the time of harvest (48 hours). For treatment with genotoxic stress, cells were incubated with 2 μg/ml of ADR for 2 h and then cultured in fresh medium until the indicated time.

### cDNA microarray and its data processing

Gene expression analysis was performed using a SurePrint G3 Human GE 8×60K microarray (Agilent, Santa Clara) according to the manufacturer’s protocol. Briefly, MCF10A *p53*+/+ and MCF10A *p53*−/− cells were treated with 0.5 μg/ml of ADR for 2 hours, then cultured with fresh medium by indicated time at 37°C. At 0 h, 12 h, 24 h and 48 h after ADR treatment, total RNA was isolated from the cells using RNeasy Plus Universal Mini Kits (Qiagen) according to the manufacturer’s instructions. Each RNA sample was labeled and hybridized to array slides. For the selection of p53-repressed gene candidates, we filtered 47,534 peaks (derived from 22,276 genes) according to the following criteria for quantification of the mRNA abundance changes: (A) calculation of the median expression in ADR-treated MCF10A *p53*+/+ cells collected at 12 h (W12), 24 h (W24), and 48 h (W48) after the treatment; (B) calculation of minimum expression in following datasets: no treated MCF10A *p53*+/+ cells (0 h, W0) and all MCF10A *p53*−/− cells datasets (K0, K12, K24, K48). As final p53-repressed gene candidates, genes whose (A)/(B) ratio < 0.5 were extracted. The MCF10A microarray data is available from the NCBI GEO database (GSE98727).

### Mice and X-ray treatment

*p53*−/− mice were provided from the RIKEN BioResource Center. Genotypes were confirmed by PCR analysis. All mice were maintained under specific pathogen-free conditions and were handled according to the Guidelines for Animal Experiments of the Institute of Medical Science (University of Tokyo, Tokyo, Japan). *p53*+/+ and *p53*−/− mice were X-ray-irradiated using the MBR-1520R-3 system (Hitachi). At 24 h after irradiation (10 Gy, total body irradiation), 24 tissues were collected from mice. The age and gender of mice were as follows: Bladder, Bone marrow, Cerebrum, Colon, Esophagus, Eyeball, Heart, Kidney, Liver, Lung, Muscle, Seminal vesicle, Small intestine, Spleen, Stomach, Testis, Thymus, and Tongue: male, 6 weeks, *n* = 3 each, Bone: male, 1 week, *n* = 3 each, Uterus: female, 10 weeks, *n* = 3 each, Mammary gland and Ovary: female, 10 weeks, *n* = 2 each. Tissues were preserved in RNAlater solution (QIAGEN) at 4°C until RNA purification. Bone marrow was resolved in RLT plus reagent provided by the RNeasy Plus Mini Kit (QIAGEN) and homogenized using a QIAshredder column (QIAGEN). The lysates were stored at −80°C until RNA purification.

### RNA sequencing and its data processing

Experimental details are described in [50]. Tissues were homogenized in QIAzol lysis reagent (QIAGEN) using Precellys 24 (Bertin Corporation). Total RNA was recovered using the RNeasy Plus Universal Mini Kit (QIAGEN). For RNA extraction from bone marrow, we used the RNeasy Plus Mini Kit (QIAGEN). We selected 256 samples for RNA sequencing analysis based on RNA quality and quantity, which were evaluated using a Bioanalyzer (Agilent) and NanoDrop spectrophotometer (Thermo Scientific). High-quality RNA was subjected to polyA+ selection and chemical fragmentation, and a 100-200 base RNA fraction was used to construct complementary DNA libraries according to Illumina’s protocol. RNA-seq was performed on a HiSeq 2500 using a standard paired-end 101-bp protocol. We used a tophat+cufflinks pipeline to process raw RNA-seq data. Before data processing, the quality of data was confirmed using FastQC. To quantify gene and transcript expression levels for all samples, we first aligned 101 bp paired-end reads to the mouse reference genome mm9/GRCm37 using Tophat (v2.0.9). The mapping parameters follow the default setting in the Tophat. After read mapping, the transcript and gene expression levels, which are represented by FPKM values, were calculated by Cufflinks (v2.2.1). For the selection of p53-repressed gene candidates, we filtered 23,282 genes according to the following criteria for quantification of the mRNA abundance changes after adding a count of one as a pseudocount: (A) calculation of the average expression in X-ray-irradiated *p53*+/+ mice (WX); (B) calculation of the minimum expression in the following datasets: average expression in no irradiated *p53*+/+ mice (W), average expression in X-ray-irradiated *p53*−/− mice (KX), and average expression in no irradiated *p53*−/− mice (K). As final p53-repressed gene candidates, genes whose (A)/(B) ratio < 0.5 were extracted. The raw data obtained in this study can be accessible in DDBJ database (http://www.ddbj.nig.ac.jp/index-e.html) with accession number of DRA005768 with bioproject accession number of PRJDB5738.

### Data processing of breast cancer patients in the TCGA database

The mRNA expression of genes, *p53* mutation status, and clinical data were obtained from The Cancer Genome Atlas (TCGA) [12]. For the selection of p53-repressed gene candidates, 1,093 breast cancer samples in the TCGA-BRCA dataset were divided into two groups according to the p53 mutation status (wild-type *p53*: *n* = 795; *p53* mutation: *n* = 298) and the average expression of each 20,475 genes was calculated. As final p53-repressed gene candidates, genes whose average expression in tumors harboring wild-type *p53* was significantly (*p* < 0.05) lower than that in tumors harboring *p53* mutation were extracted.

### Quantitative real-time PCR

Total RNA was isolated from human cells using RNeasy Plus Mini Kits (Qiagen) and RNeasy Plus Universal Mini Kits (Qiagen) according to the manufacturer’s instructions. Complementary DNAs were synthesized using Super Script III reverse transcriptase (Invitrogen). Quantitative real-time PCR (qPCR) was performed using the SYBR Green Master Mix with a Light Cycler 480 instrument (Roche). For ΔΔCt method, *β-actin* was used as a reference gene. Fold expression change was calculated as 2-ΔΔCt according to the manufacturer’s protocol. Primer sequences are listed in Supplementary Table S5.

### Western blot analysis

Total cell lysates were prepared with lysis buffer containing 50 mM Tris-HCl (pH 7.5), 100 mM NaCl, 1% NP-40, Protease Inhibitor Cocktail Set III (Calbiochem), and phosphatase inhibitor cocktail set II (MERCK MILLIPORE) and normalized by protein concentration using the BCA method (Thermo Scientific). The protein samples were separated on SDS-polyacrylamide gel electrophoresis and transferred to nitrocellulose membranes (Hybond™ ECL™, Amersham). Membranes were blocked in TBS-T containing 5% nonfat milk for 1 hour at room temperature. Then, the membranes were incubated with primary antibodies according to the manufacturer’s instructions for 18 hours at 4°C. After that, the membranes were incubated with horseradish peroxidase-conjugated goat anti-mouse IgG (Santa Cruz Biotechnology) and visualized by chemiluminescent detection (Immobilon, Millipore).

### Statistical analysis

Statistical analysis was performed using an unpaired two-tailed Student’s *t*-test. The F-test was used to determine whether variances were equal or unequal. 1,093 patients in TCGA-BRCA dataset from TCGA were subjected for the statistical analysis. Survival analysis was performed by Kaplan-Meier method using EZR (v.1.27) software. Hazard ratio was calculated by using EZR (v.1.27) software. Fisher’s exact test was performed using EZR (v.1.27) software. A *p*-value of < 0.05 was considered statistically significant.

## SUPPLEMENTARY FIGURES AND TABLES












