# Supplementary material for: Identification of a p53-repressed gene module in breast cancer cells
Source: Oncotarget. 2017 Jul 26;8(34):55821–36. doi: 10.18632/oncotarget.19608 (PMC5593526; doi:10.18632/oncotarget.19608)
Supplement: Supplementary file 1 [file oncotarget-08-55821-s001.pdf]

Identification of a p53-repressed gene module in breast cancer cells

SUPPLEMENTARY MATERIALS

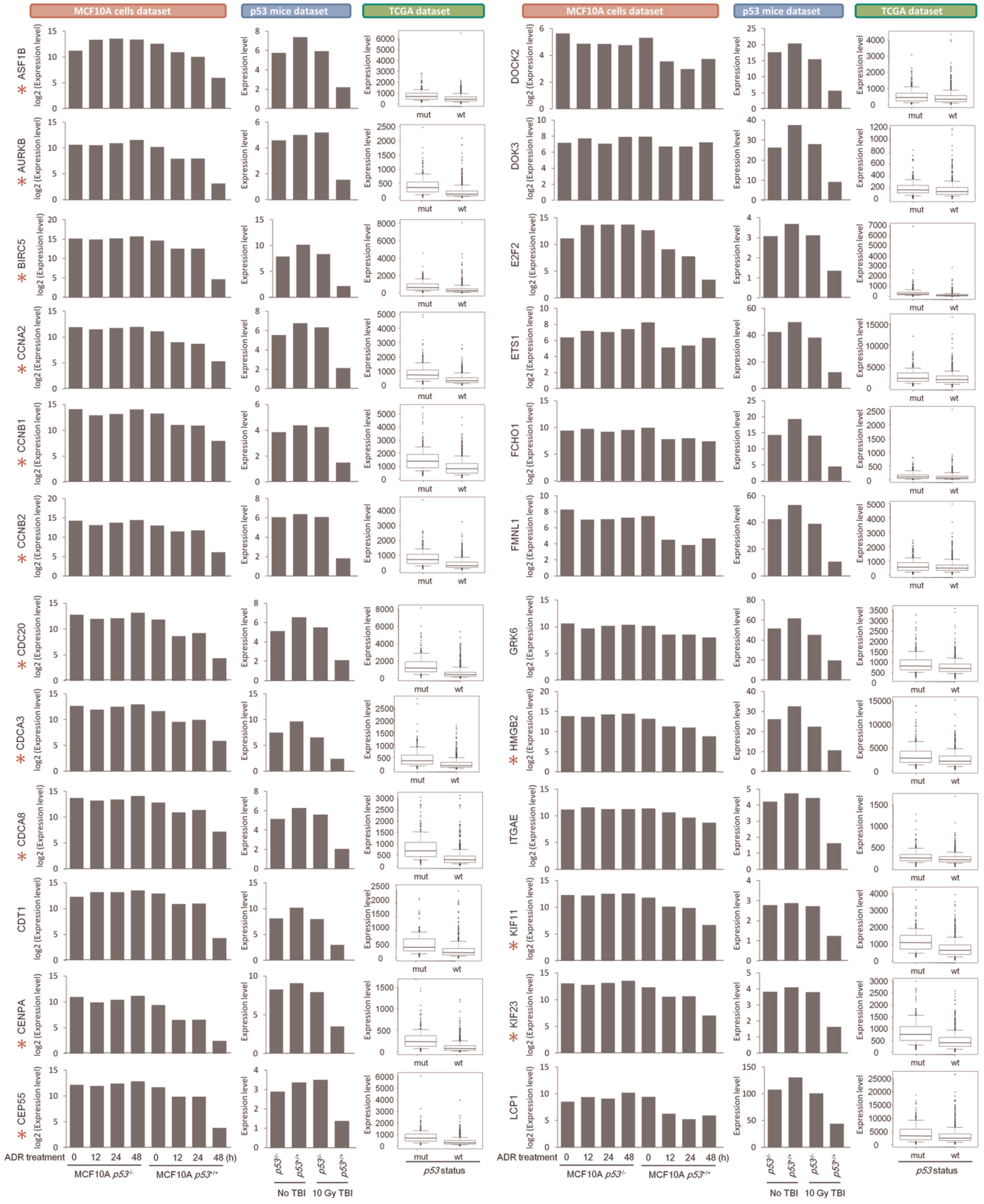

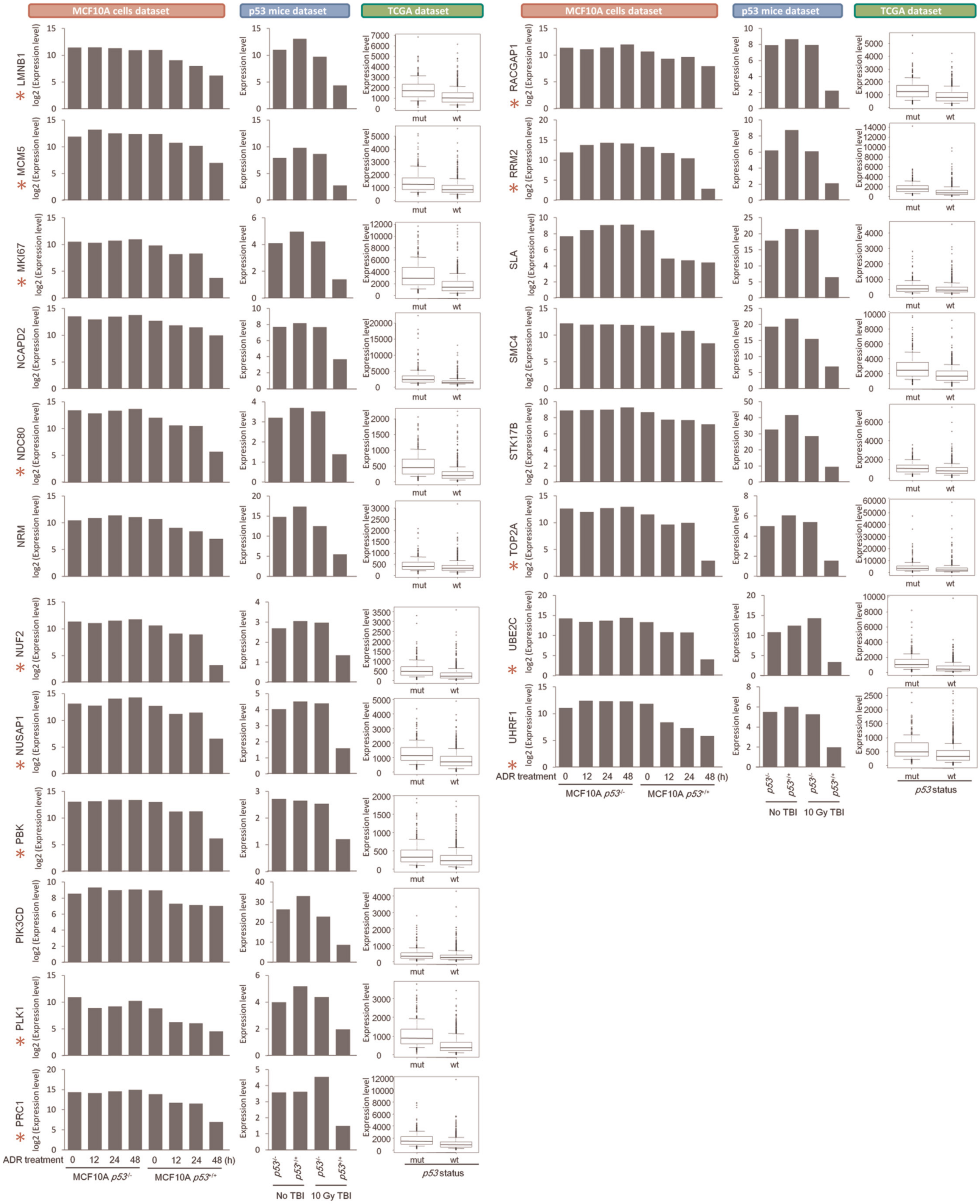

Supplementary Figure 1: Identification of 44 p53-repressed gene candidates. The results obtained from the MCF10A cells dataset (Figure **1A**), *p53* mice dataset (Figure **1B**), and TCGA dataset (Figure **1C**) of 44 p53-repressed gene candidates are shown. *: p53-repressed gene module.

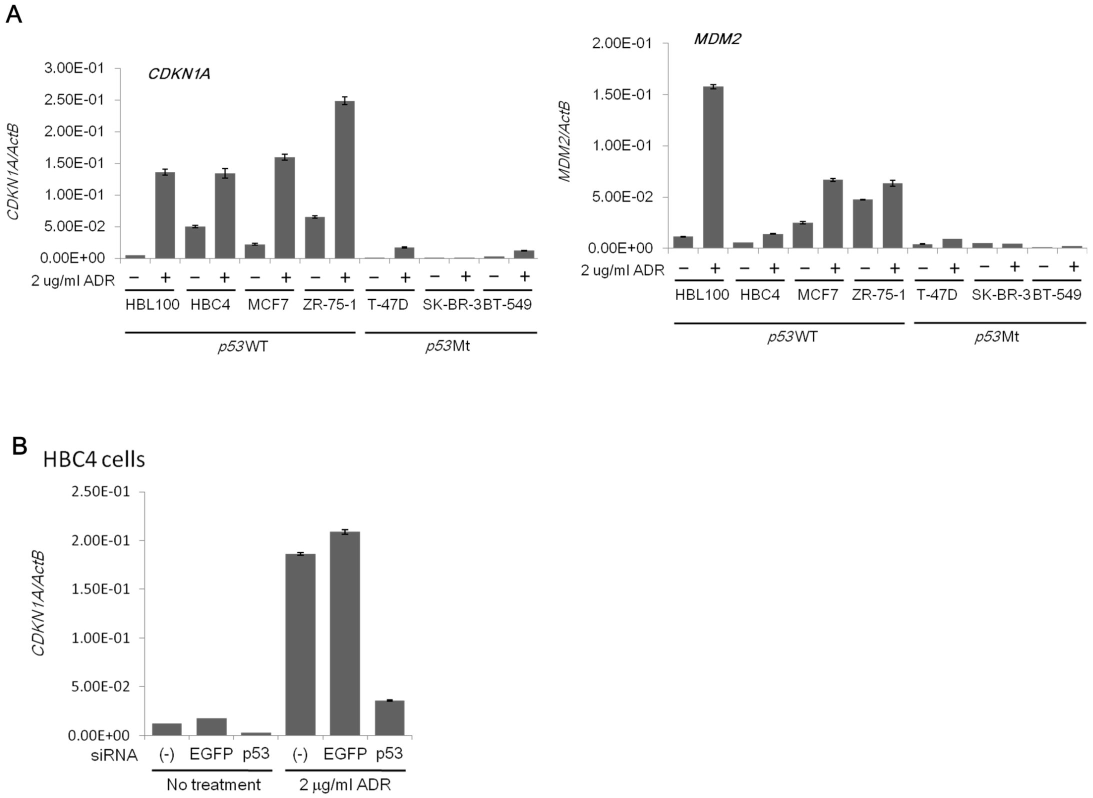

Supplementary Figure 2: The expression dynamics of p53-induced genes under genotoxic stress condition. (**A**) Indicated breast cancer cells were treated with 2 μg/ml ADR for 2 hours. Forty-eight hours after treatment, the expression level of *p21/CDKN1A* (left) and *MDM2* (right) was determined using qPCR analysis. The data are normalized against β-actin and presented as the mean±SD from three independent experiments. (**B**) At 24 hours after transfection of indicated siRNA, HBC4 cells were treated with 2 μg/ml ADR for 2 hours. Forty-eight hours after treatment, the expression level of *p21/CDKN1A* was determined using qPCR analysis. The data are normalized by β-actin and presented as the mean±SD from three independent experiments.

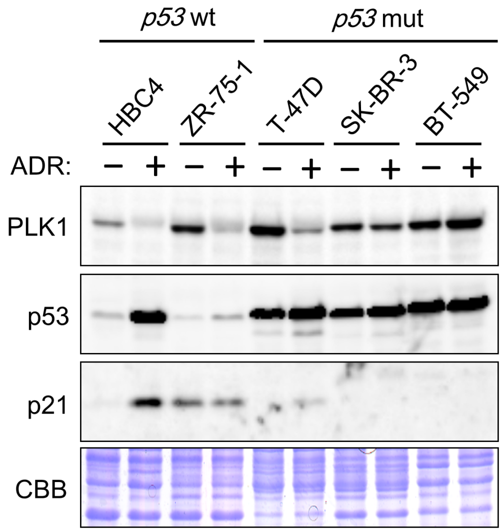

Supplementary Figure 3: Effect of ADR on the regulation of PLK1 expression. Indicated breast cancer cells were treated with 2 μg/ml ADR for 2 hours. Forty-eight hours after treatment, the expression level of indicated proteins was examined by western blotting.

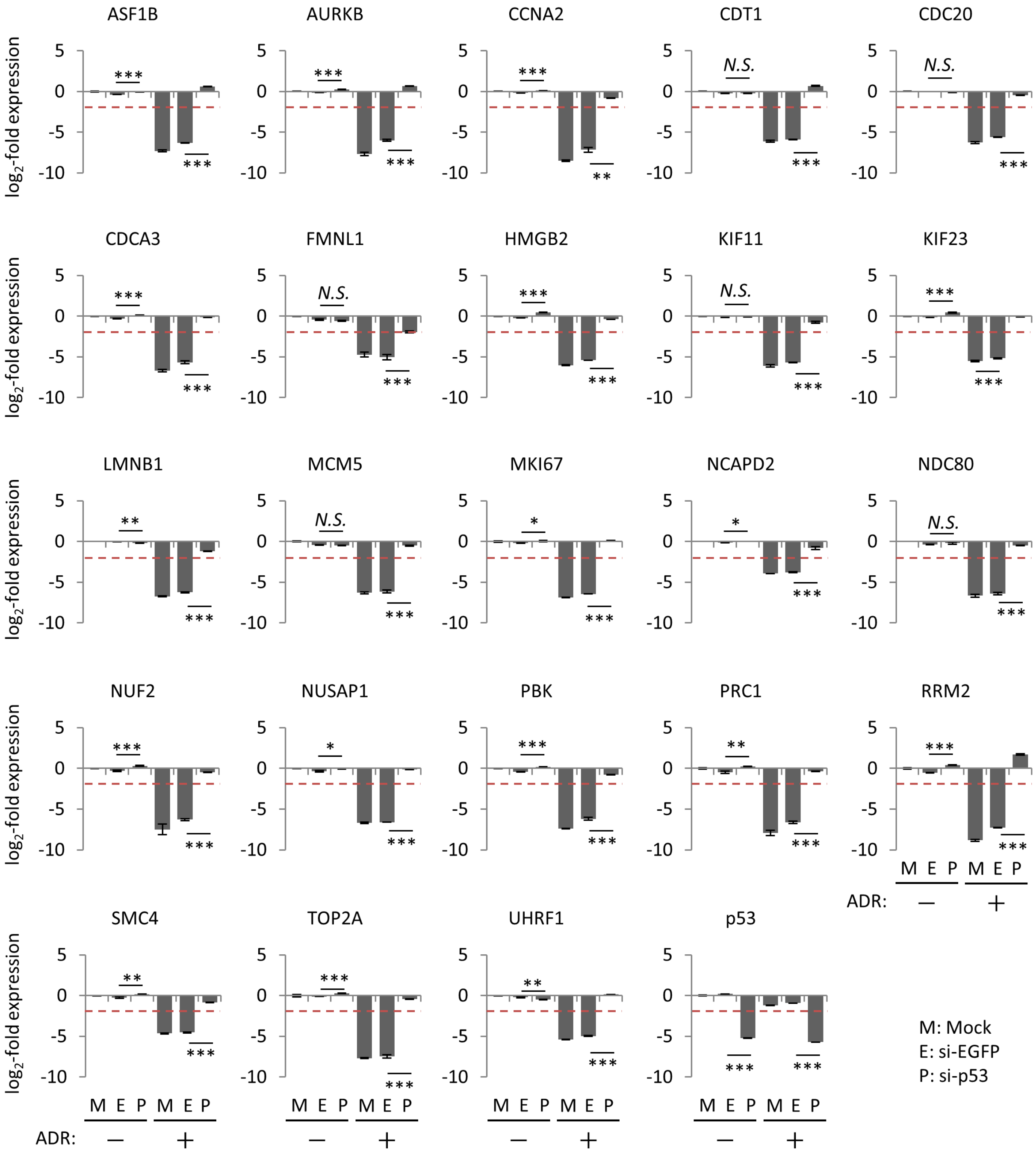

Supplementary Figure 4: Effect of p53 knockdown on the regulation of common p53-repressed genes. Additional data from Figure 3 are shown. The data are presented as the mean±SD from three independent experiments. The red dotted line shows cutoff value. *P<0.05, **P<0.01, ***P<0.001, *N.S.*, not statistically significant.

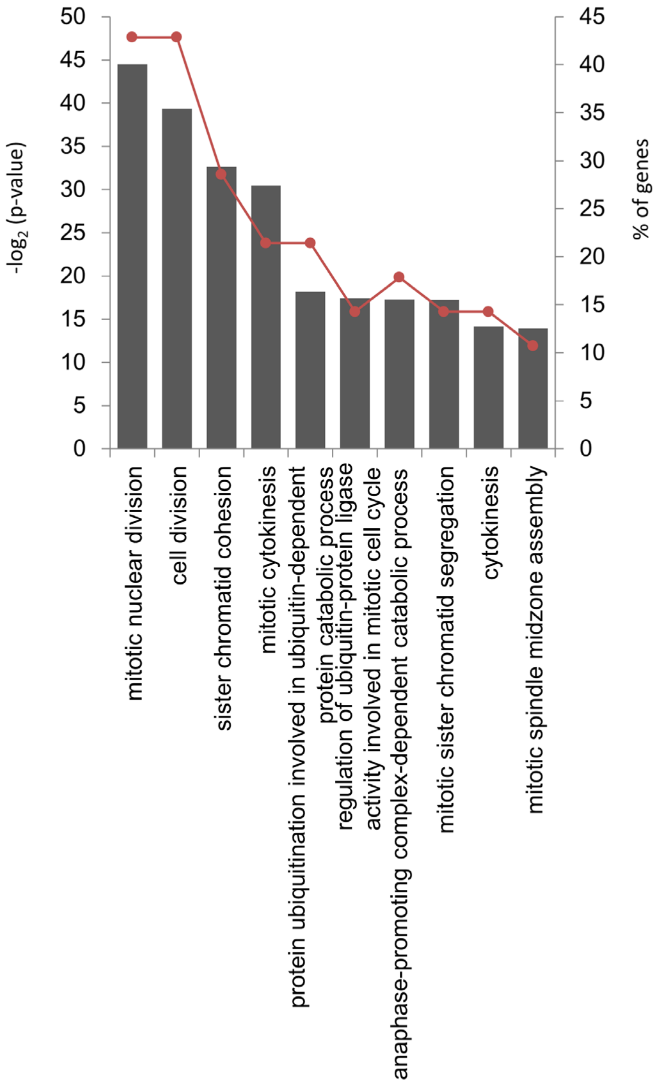

Supplementary Figure 5: Gene ontology analysis of the p53-repressed gene module. Gene ontology analysis by DAVID Functional Annotation tool is shown. Bar: -$\log_2$ (*P*-value). Polygonal line: % of the p53-repressed gene module elements included.

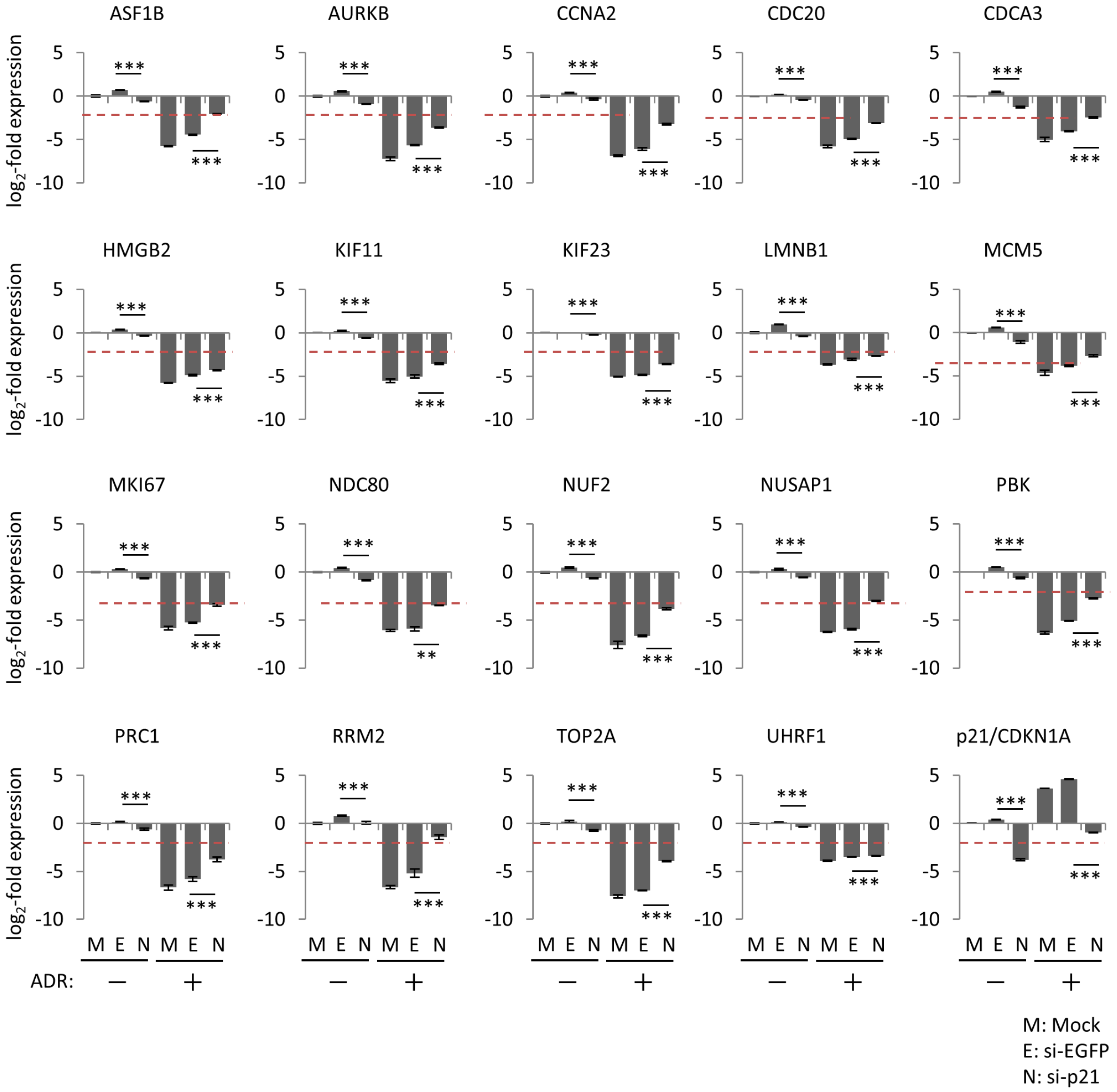

Supplementary Figure 6: Effect of p21/CDKN1A knockdown on the regulation of p53-repressed gene module. Additional data from Figure 7 are shown. The data are presented as the mean±SD from three independent experiments. The red dotted line shows the cutoff value. **P<0.01, ***P<0.001, *N.S.*, not statistically significant.

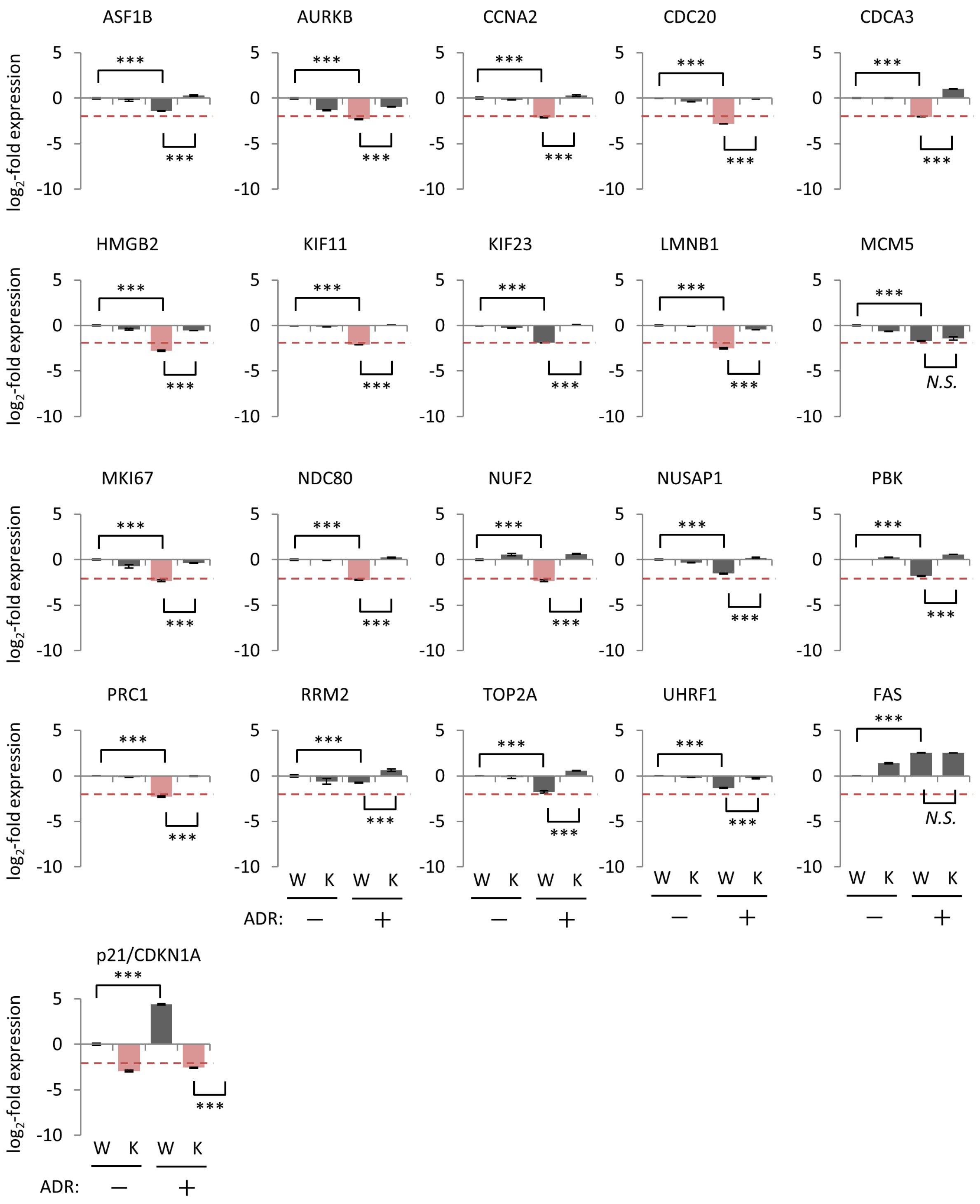

Supplementary Figure 7: Effect of p21/CDKN1A knockout on the regulation of p53-repressed gene module. Additional data from Figure 8 are shown. The data are presented as the mean±SD from three independent experiments. The red dotted line shows the cutoff value. Red graph: lower than cutoff value. ***P<0.001, *N.S.*, not statistically significant.

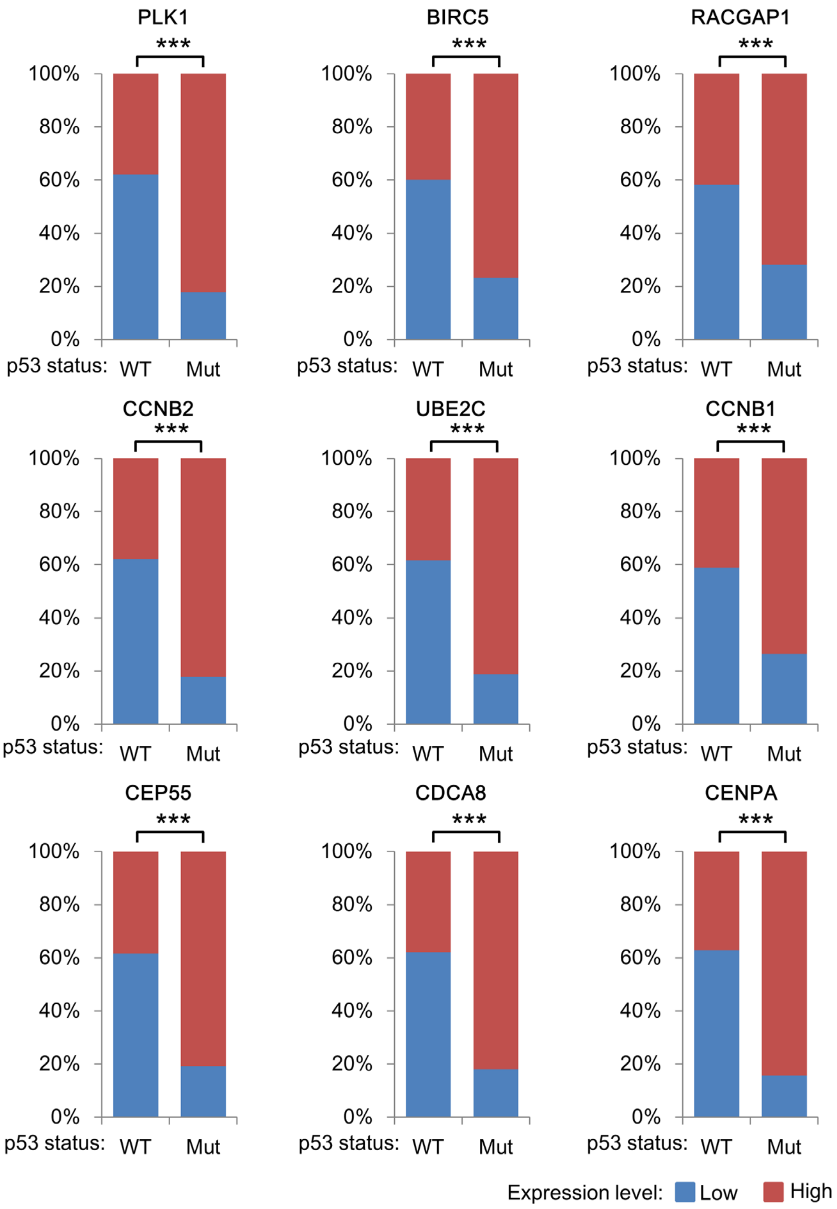

Supplementary Figure 8: Correlation between the expression levels of p53-repressed genes and *p53* status. The proportion of high and low level expression groups for each gene in breast invasive carcinoma patients harboring either wild-type p53 or mutated p53 is shown. As presented in Figure 9, the breast cancer patients were subdivided into high (n = 546) or low (n = 547) groups according to the expression level of the indicated genes (cutoff: the median mRNA expression level of each gene). Breast cancer patients harboring wild-type p53; n = 795. Breast cancer patients harboring mutated p53; n = 298. ***: Fisher's exact test, $P < 0.001$.

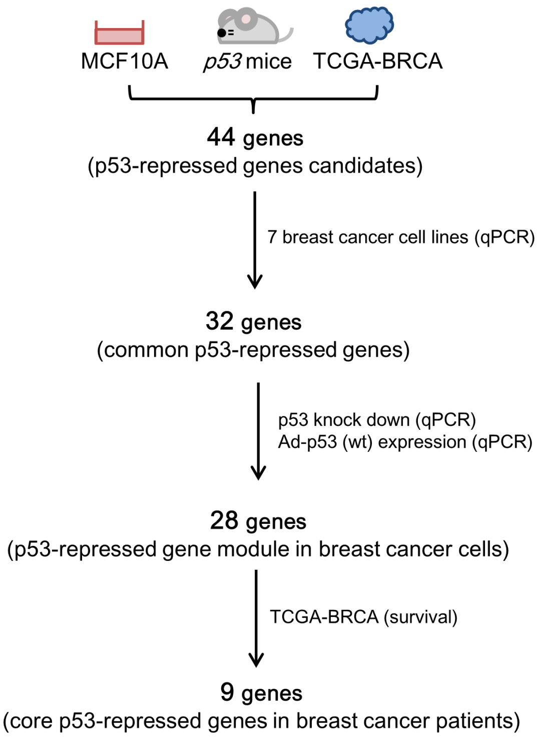

Supplementary Figure 9: Schematics of p53-repressed gene module screen. Schematics of our screen are illustrated.

Supplementary Table 1: Transcriptome data obtained from ADR-treated MCF10A p53$^{+/+}$ cells and p53$^{-/-}$ cells

Supplementary Table 2: Transcriptome data obtained from X-ray-irradiated p53$^{+/+}$ mice and p53$^{-/-}$ mice

Supplementary Table 3: Transcriptome data obtained from BRCA harboring wild-type p53 and p53 mutations in TCGA dataset

Supplementary Table 4: A list of p53-mediated genes

Supplementary Table 5: Primer sequences used for qPCR analysis

For Supplementary Tables see in Supplementary Files.
